# Preclinical evaluation of Raman spectroscopy for pedicular screw insertion surgical guidance in a porcine spine model

**DOI:** 10.1117/1.JBO.28.5.057003

**Published:** 2023-05-31

**Authors:** Ivan Kosik, Frédérick Dallaire, Layla Pires, Trang Tran, Frédéric Leblond, Brian Wilson

**Affiliations:** a Princess Margaret Cancer Center, Toronto, Ontario, Canada; b Polytechnique Montreal, Department of Engineering Physics, Montreal, Quebec, Canada; c Centre de recherche du Centre hospitalier de l’Université de Montréal, Montreal, Quebec, Canada; d Institut du cancer de Montréal, Montreal, Quebec, Canada

**Keywords:** Raman spectroscopy, orthopedic, surgery, guidance, supervised machine learning, artificial intelligence, pedicle screw

## Abstract

**Significance:**

Orthopedic surgery is frequently performed but currently lacks consensus and availability of ideal guidance methods, resulting in high variability of outcomes. Misdirected insertion of surgical instruments can lead to weak anchorage and unreliable fixation along with risk to critical structures including the spinal cord. Current methods for surgical guidance using conventional medical imaging are indirect and time-consuming with unclear advantages.

**Aim:**

The purpose of this study was to investigate the potential of intraoperative *in situ* near-infrared Raman spectroscopy (RS) combined with machine learning in guiding pedicular screw insertion in the spine.

**Approach:**

A portable system equipped with a hand-held RS probe was used to make fingerprint measurements on freshly excised porcine vertebrae, identifying six tissue types: bone, spinal cord, fat, cartilage, ligament, and muscle. Supervised machine learning techniques were used to train—and test on independent hold-out data subsets—a six-class model as well as two-class models engineered to distinguish bone from soft tissue. The two-class models were further tested using *in vivo* spectral fingerprint measurements made during intra-pedicular drilling in a porcine spine model.

**Results:**

The five-class model achieved >96% accuracy in distinguish all six tissue classes when applied onto a hold-out testing data subset. The binary classifier detecting bone versus soft tissue (all soft tissue or spinal cord only) yielded 100% accuracy. When applied onto *in vivo* measurements performed during interpedicular drilling, the soft tissue detection models correctly detected all spinal canal breaches.

**Conclusions:**

We provide a foundation for RS in the orthopedic surgical guidance field. It shows that RS combined with machine learning is a rapid and accurate modality capable of discriminating tissues that are typically encountered in orthopedic procedures, including pedicle screw placement. Future development of integrated RS probes and surgical instruments promises better guidance options for the orthopedic surgeon and better patient outcomes.

## 1 Introduction

Orthopedic surgery has become commonplace in most industrialized countries with an increasing range of techniques and patients.^1^^,^^2^ Given growing life expectancy and concomitant need to maintain an active lifestyle, more patients are electing to have orthopedic procedures, including, for example, decompression with spinal fusion.^3^^–^^5^ In this procedure, precise placement of intramedullary screws represents the most critical aspect and requires many years of surgical experience due to the inherent complexity and variability of the tasks involved.^6^^–^^8^ Placement requires that all screws be positioned into a narrow channel within the vertebral pedicle, with a tolerance of only a few millimeters to prevent bone breach and to limit the risk of nerve or spinal cord damages. As a result, clinicians must have a high level of confidence during screw placement and, since spinal fusion often requires installation of dozens of screws, reducing the time needed for each placement could have a major impact on the surgical workflow. Hence, new technologies that enable rapid and effective surgical guidance during screw placement are critical to minimize complications and surgery time.^9^

In addition to preoperative radiological imaging, spine surgeons currently rely on guidance using x-ray fluoroscopy and/or ultrasound imaging, both of which have significant limitations, including two-dimensional (rather than three-dimensional) imaging, and ionizing radiation doses to the patient and operating room personnel in the case of fluoroscopy.^10^^–^^12^ Moreover, clinical ultrasound systems are plagued with poor spatial resolution and shadowing artifacts. Both fluoroscopy and ultrasound imaging also require co-registration with radiographic imaging, further complicating the procedures.^11^ Intraoperative x-ray computerized tomography (CT) can offset some of these deficiencies but is time consuming and adds to the radiation dose to the patient.^13^. Emerging robotic-assisted surgery is promising but introduces procedural complexities, increases cost, and requires advanced operating-room staff training.^6^^,^^9^ As a result of those limitations, there is an unmet need for direct real-time spine surgery guidance technology capable of dynamically informing the surgeon about tissue type in the local vicinity of the surgical instruments to limit adverse effects during screw placement.

A minimum level of direct guidance could be provided by a system that differentiates vertebral bone from soft tissues, especially the spinal cord as the most critical structure at risk.^14^^,^^15^ In principle, successful placement of pedicle screws could be achieved by ensuring that the screws always remain within bone structures during insertion, guaranteeing a strong bond and no breach of the bone surface, thereby limiting fracture risk and damage to adjacent soft tissues. This approach has been investigated using various optical techniques, including near-infrared (NIR) spectroscopy, diffuse reflectance spectroscopy, and photoacoustic imaging.^16^^–^^18^ However, each of these relies essentially on a single predictive parameter (e.g., absorbance or elastic scattering, impedance) to differentiate bone from non-bone, which can limit the specificity of tissue identification. Moreover, these techniques can be susceptible to failure in cases where nominal specificity is degraded by, for example, intraoperative bleeding.^19^ Ideally, an orthopedic surgical guidance system should allow highly accurate discrimination of multiple tissues in real time, i.e., ∼100  ms.

A candidate technique is NIR Raman spectroscopy (RS) where the inelastic scattering of light from biological tissue reveals the vibrational modes of all molecules in the sample. Specifically, the so-called fingerprint region of RS (wavenumber shifts: ∼400 to $1800\;{\mathrm{cm}}^{-1}$) provides information relating to the primary and secondary structure of proteins (e.g., amide I and III bands, also potentially including differentiation of α-helix and β-helix conformation) as well as on vibrational bonds predominantly present in the three aromatic amino acids (phenylalanine, tryptophan, and tyrosine). The technique also sheds light on other biomolecules, leading to detectable bands associated with nucleic acids (DNA, RNA), glucose (e.g., glycogen), and different types of lipids. When implemented via fiberoptic probes, RS has a proven track record for real-time intraoperative tissue classification in other settings, both for diagnostics such as tumor detection and for surgical guidance such as in the brain and prostate.^20^^–^^24^

Several research groups have investigated RS in orthopedics. For example, Shaikh et al.^25^ showed that RS could specify cartilage injury type and so aid in clinical treatment planning,while Pavlou et al.^26^ demonstrated RS sensitivity to changes associated with onset of osteoarthritis. Moreover, Buckley et al.^27^ detected RS signatures of bone mineralization, indicating statistically significant predictors of fragility fractures in cadaveric specimens: while the corresponding first attempts *in vivo* were statistically underpowered, planned system improvements looked promising. Fraulob et al.^28^ targeted orthopedic implant surgery, using intraoperative RS to assess the bone-implant interface. For a wider perspective, Fosca et al.^29^ have published a comprehensive review of RS applied to skeletal disorders.

Practical implementation of RS guidance for placing pedicle screws could take the form of fiberoptic sensors mounted at the tip of a Kirshner wire, trocar, drill bit, screws, or other intramedullary rods.^16^^,^^30^ Such technology integration is in progress by us and other groups developing, for example, low-noise rotary fiber optic joints.^31^ To maximize the potential utility of RS in spinal surgery, the present study focuses on measuring the Raman spectral fingerprints of all tissue types in and around the vertebrae, using a hand-held fiber optic probe system from the company reveal surgical that is based on a laboratory prototype developed by our group.^24^^,^^32^ The study was designed to evaluate the potential of intraoperative RS for guiding spinal fusion surgeries and pedicle screw placement. All experimental protocols were performed in a normal swine model that has similar size, composition, and morphology to humans.^33^

## 2 Materials and Methods

### 2.1 Study Design

The overall study comprised, first, measurements of the Raman spectra of bone and soft tissues in freshly excised swine vertebra *ex vivo*. Those measurements were used as input data to a supervised machine learning support vector machine (SVM) analysis that associated each tissue type with the corresponding Raman spectral fingerprint. This resulted in a trained and validated multi-class predictive model designed to discriminate between six tissue types: bone, cartilage, fat, ligament, muscle, and spinal cord (model I). The *ex vivo* measurements were also used to produce two-class models trained and validated to discriminate between bone and spinal cord (model II) and between bone and all soft tissue types (Model III). The predictive accuracy (sensitivity and specificity) of all tissue classifiers was tested using an independent hold-out dataset that was not used during the model training/validation phase.

To emulate real-world conditions met during orthopedic procedures, the two-class models were independently tested on two other Raman spectral fingerprint datasets. One set was acquired *in situ* from freshly excised vertebra, while the other was acquired *in vivo* under conditions closer to the real-world surgical scenario. In both cases, the spectroscopic data were acquired during bone drilling using the RS probe.

### 2.2 Raman Spectroscopy System and Measurement Protocol

**Fig. 1:**
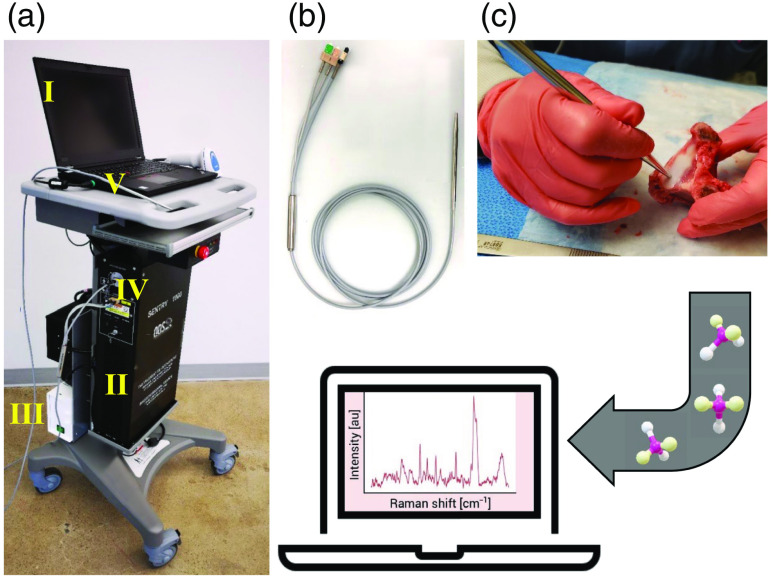
RS system. (a) Photograph of the reveal system with major components annotated: (I) laptop with graphical user interface, (II) illumination and detection module housing, (III) hospital-grade isolation transformer, (IV) laser and spectrometer fiberoptic connection panel, and (V) fiberoptic bundle with optical connectors and hand-held probe. (b) Close up of the probe in contact with a vertebral tissue sample. (c) Example Raman spectrum.

The system used to obtain tissue spectral fingerprints (Sentry 1000-R, Reveal Surgical Inc., Montreal, Canada) is an NIR RS instrument that has been approved for investigational clinical testing in neurosurgery by Health Canada. Mounted on a portable cart, it consists of illumination and detection modules, a hand-held probe, and a laptop computer (Fig. 1).

The illumination module houses a 785 nm diode laser (class IIIB) with a maximum output of 100 mW (Innovative Photonic Solutions, Plainsboro, New Jersey). The detection module comprises an NIR spectrometer and associated optical and electronic components. The spectrometer consists of a charge-coupled device sensor (Newton model, Andor Technology, Belfast, United Kingdom) cooled to −40°C, a 100  μm spectrometer slit, and a diffraction transmission holographic grating. The probe has a central light-excitation fiber surrounded by nine collection fibers (100  μm core diameter) potted within a stainless-steel ferule and terminated with a tapered tip and interface window. The probe is connected to the laser and the spectrometer through a 3 m long fiberoptic cable. It is sterilizable, reusable, and has the shape of a 12 cm long stylet. Where the probe contacts tissue, there is a conical tip of outer diameter 2.1 mm. Optical filters are mounted within the probe tip to minimize signals from the fiber materials and the tissue autofluorescence. The spectrometer has high sensitivity across the range 400 to $2000\;{\mathrm{cm}}^{-1}$, with an average resolution of $1.8\;{\mathrm{cm}}^{-1}$. A converging lens at the tip of the probe ensures contact measurements interrogated a 0.5 mm diameter spot. The system is controlled by proprietary software (Reveal Surgical Inc., Montreal, Canada) that allows acquisition parameters to be set by the user, including laser power, exposure time per spectrum, and number of repeated measurements (i.e., accumulations) at each point. A laboratory version of the system has been used in several clinical studies for intraoperative tumor resection guidance in various organs including breast,^34^ brain,^24^^,^^35^^,^^36^ ovaries,^37^ and prostate.^23^^,^^38^^–^^40^

System calibration comprised acquisition on an NIST Raman standard (SRM 2214) to correct for the instrument response.^41^ The probe tip was cleaned with isopropyl alcohol before and after calibration, as well as throughout the study to minimize cross-contamination of signals from previously measured tissues. For each spectral measurement, a suitable illumination exposure time was selected based on the optical properties of the tissue under investigation to ensure maximum photon counts without saturating the detection electronics. Once a measurement site was selected, the tip of the probe was held in contact with the tissue at a right angle to maximize optical coupling [Fig. 1(c)]. Two individuals participated in the measurements, one preparing the animal model and tissues and the other operating the system, thereby minimizing potential motion artifacts during each measurement. All measurements were carried out under low ambient lighting to minimize background noise. At least three spectral fingerprints were collected at each measurement location (i.e., accumulations), using total integration times ranging from 0.4 to 20 s per spectrum, depending on the tissue type. For example, typical integration times per accumulation for bone and spinal cord were 1.0 and 0.8 s, respectively.

### 2.3 Animal Protocol

All animal studies were carried out under institutional review and approval (AUP #6578: University Health Network, Toronto, Canada). To minimize the number of swine needed to meet the study objectives, animals that were already enrolled in other approved terminal studies were used for the* ex vivo* and *in situ* measurements. The *in vivo* study was performed only after these prior data demonstrated high and consistent results. The animals were free from genetic manipulation, tracer probes or any other exogenous substances except anesthesia. The 33 kg male Yorkshire swine was fasted 8 h prior to surgery and received meloxican IM (0.4  mg/kg) preoperatively. General anesthesia was induced and maintained with inhaled isoflurane, 4% and 2.5%, respectively. During the entire procedure, the animal had all vital signs monitored closely and received buprenorphine IM (0.4  mg/kg) and fluids i.v. After the procedure, the animal was euthanized by intravenous injection of KCl (150  mg/kg).

### 2.4 *Ex Vivo* Spectroscopic Measurements

**Fig. 2:**
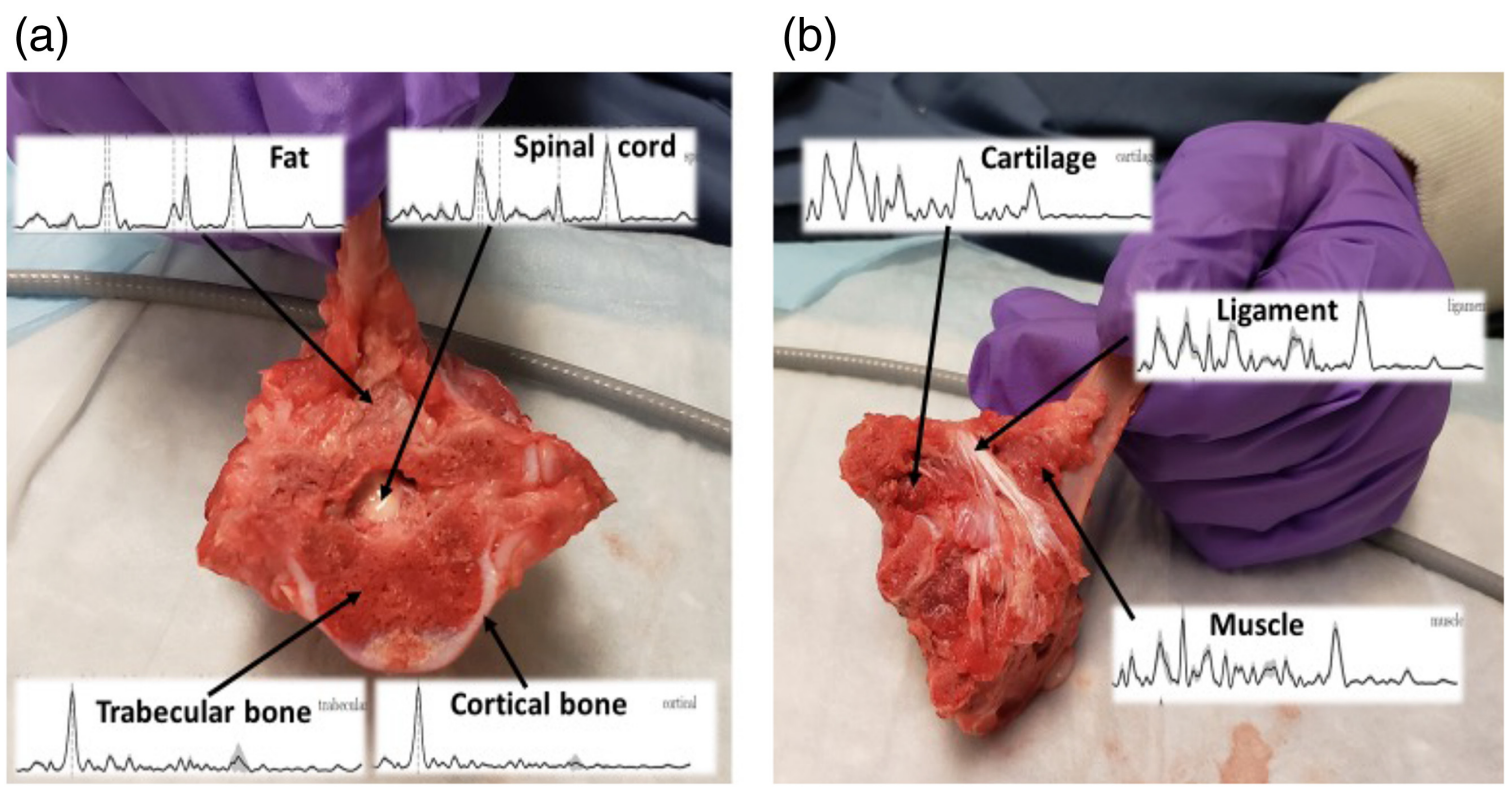
Example of freshly excised vertebra in (a) axial and (b) lateral views, with the major components and Raman spectral fingerprints labeled.

The spinal columns of three swine were cut with a surgical bone saw to remove one thoracic vertebra, along with intact soft tissues that interface with the bone. Figure 2 is an example, showing the cortical and trabecular bone (henceforth lumped together into the “bone” category) and soft tissues (cartilage, fat, ligament, muscle, and spinal cord). Prior to collection of the Raman spectra, the sample was lightly rinsed with saline to help identify the structures visually and minimize spectral crosstalk from blood and cerebrospinal fluids. On average, eight different measurement sites were selected per tissue type on each vertebra, contingent on availability of suitable exposed tissue surfaces. The tissue class was identified based on visual inspection, with knowledge of spinal anatomy.

### 2.5 *In Situ* and *In Vivo* Spectroscopic Tissue Measurements

**Fig. 3:**
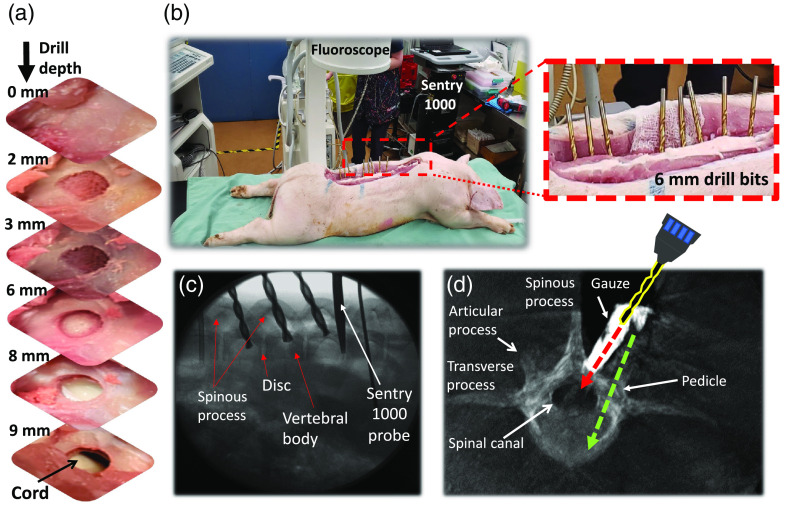
(a)–(c) *In situ* and (d) *in vivo* drilling and RS procedures. (a) Photographs at each stage of drilling demonstrating mm-level depth control and showing the bone surface and the drilling cavity at the cortical/trabecular interface (2 mm), trabecular bone (3 mm), trabecular/cortical interface (6 mm), epidural space (8 mm), and spinal cord breach (9 mm). (b) *In situ* drilling under fluoroscopy imaging guidance with a labeled radiograph shown in (c). (d) 3D CT image guidance in the axial view showing labeled vertebra with mis-directed drill direction toward spinal canal (red arrow), indicated by insertion of contrast-infused gauze. The green arrow indicates the correct drill trajectory through the pedicle.

*In situ* spectra were acquired for each tissue category (bone, cartilage, fat, ligament, muscle, and spinal cord) in two freshly sacrificed animals. Spinal surfaces were reached using a conventionally open long-segment incision technique for pedicle access prior to drilling in the vertebra. The bone surface was exposed so that tissues could be visually identified. In total, 14 holes were drilled from four different vertebrae in each animal [Figs. 3(a)–3(c)]: eight lumbar, five thoracic, and two cervical. The last was the most difficult to access due to a thick overlaying muscle mass. Overall, this resulted in 50 spectral measurements in one animal and 38 spectra in the other.

Following *in situ* testing, *in vivo* measurements were performed in one anesthetized animal to confirm the system and machine-learning model performance under more clinically realistic conditions [Fig. 3(d)]. Four vertebrae in total were examined from thoracic, lumbar, and cervical sections of the spine. Since a critical requirement during spinal fusion surgery is to ensure proper placement of pedicle screws, detection of misdirected screw trajectories resulting in spinal canal breaches was a primary focus during trajectory planning. The *in vivo* measurements resulted in 44 spectral fingerprints acquired along four different trajectories, one trajectory per vertebra: three vertebrae had 12 spectral measurements, and one had eight measurements.

All *in situ* and *in vivo* measurements were performed by intra-pedicular drilling in 1 mm steps using a cordless drill equipped with a shaft-collar drill-stop mounted on a 6 mm titanium drill bit with a 135 deg drill point angle. Cooling and lubrication were provided by saline rinsing of the drilling cavity at each step prior to measurement of the spectral fingerprint. The drill stop provided mm-level depth control, permitting precision measurement of spectra from tissue layers and layer interfaces. *In situ* guidance during drilling was carried out as shown in Fig. 3(c) under x-ray fluoroscopy (OEC 9800, GE Healthcare). The *in vivo* drilling was guided using 3D CT (Cios Spin C-arm, Siemens Healthcare Ltd.), as shown in Fig. 3(d), where a (deliberate) spinal canal breach was indicated using contrast medium-soaked gauze pressed into the drilling cavity.

### 2.6 Data Pre-Processing and Raman Spectral Fingerprint Interpretation

The following data pre-processing steps were applied to each spectroscopic measurement:^41^ (1) subtraction of a dark count background measurement acquired with the laser turned off prior to each repeat acquisition (e.g., to remove residual contamination from ambient light sources), (2) x-axis (wavenumber) normalization and instrument response correction from spectral measurements acquired in calibration materials (acetaminophen powder and NIST 785 nm Raman standard, respectively), (3) curve smoothing using a Savitzky-Golay filter of order 3 with a window size of 11 (unit-less optimization parameter), (4) averaging of successive measurements acquired at the same location, (5) baseline subtraction using a custom algorithm, BubbleFill.^41^ Finally, the resulting Raman spectral fingerprints were normalized based on standard-normal-variate (SNV). This normalization technique implies that the “intensity” associated with each spectral bin or band must be interpreted as a variation relative to the average of all detected inelastic scattering contributions across the spectral domain.

Recent RS studies have contributed reliable and well-characterized spectra associated with specific tissues. Raman peaks are narrow (typically $<50\;{\mathrm{cm}}^{-1}$) and in many cases can be associated with a specific chemical bond or functional group. For example, Movasaghi et al.^22^ have compiled the most frequent reported Raman bands in tissues. For each tissue type considered here (bone, cartilage, fat, ligament, muscle, and spinal cord), the main visually distinguishable Raman bands were identified on the *ex vivo* Raman spectral fingerprints, mostly based on the Movasaghi et al. paper. The resulting band assignments are listed in Table 1 and were also cross-checked against other publications.

**Table 1:** Principle vibrational modes, band assignments, and corresponding relative concentration in each tissue type indicated by the number of asterisks from lowest (*) to highest (****).^22^ The tissue label “connective” refers to the categories “ligament” and “cartilage” combined.

Peak center (${\mathrm{cm}}^{-1}$)	Main vibrational modes	Biomolecular assignments	Tissue type				
			Muscle	Connective	Spinal cord	Fat	Bone
817	C─C stretching	Collagen	—	****	—	—	—
857	C─C vibration	Collagen backbone hydroxyproline	***	****	—	—	—
943–945	C─C stretching	Protein backbone	***	****	—	—	—
959–965	Calcium–sphospate stretch band	Calcium hydroxyapatite	—	***	—	—	****
1005	Symmetric ring breathing	Phenylalanine	***	****	—	—	—
1067–1069	Bands ν(C─O)	Proline, collagen	*	****	***	**	—
1081–1083	C─N stretching	Proteins, lipids	*	****	***	**	—
1131	C─C skeletal stretching	Palmitic and fatty acids	****	***	**	—	—
1249	Stretching vibrations of C─N bonds	Amide III	****	—	***	—	—
1269–1271	C─H, Δ (═C─H)	Amide III, lipids, phospholipids	—	****	—	***	—
1305	${\mathrm{CH}}_{3}$, ${\mathrm{CH}}_{2}$ twisting/deformation	Collagen	**	*	***	****	—
1325	${\mathrm{CH}}_{3}$${\mathrm{CH}}_{2}$ waging mode	Nucleic acids	****	***	—	—	—
1344	δ(CH), residual vibrations	Amide III	***	****	—	—	—
1439–1442	${\mathrm{CH}}_{2}$ and ${\mathrm{CH}}_{3}$ deformation, ${\mathrm{CH}}_{2}$ bending	DNA/RNA, protein (amide I), lipids	*	**	****	***	—
1448–1451	${\mathrm{CH}}_{2}$${\mathrm{CH}}_{3}$ deformation	Collagen, lipids, proteins	****	***	**	*	—
1641	Deformation vibrational δ(HOH)	Water	—	****	—	—	—
1658–1665	ν-helix	Amide I	****	***	*	**	

### 2.7 Machine Learning Tissue Classification Models

Three different machine learning models were produced from the *ex vivo* measurements. Model I consisted of a six-class predictive model (bone versus cartilage versus fat versus ligament versus muscle versus spinal cord), while models II and III were two-class models. Model II differentiated bone from spinal cord tissue and model III discriminated bone from all types of soft tissues. All models were tested on a hold-out data subset: 60% for model training and validation and 40% for testing. Further, models II and III were independently tested using the datasets acquired under conditions closer to real-world, i.e., the *in situ* and *in vivo* datasets.

Each processed spectrum led to a Raman spectral fingerprint comprising more than 900 spectral bins. Prior to machine-learning model development, the spectra were reduced to N<20 spectral features using a linear SVM approach with L1-regularization (Lasso regression) optimization.^42^ The resulting dimensionally reduced features set—in the form of N pre-selected spectral intensities associated with specific wavenumber values—was used to train and validate the machine-learning models using a linear SVM approach with L2-regularization (Ridge regression). Both phases—feature selection and machine learning modeling—were associated with one SVM hyperparameter that is conventionally labeled C. The values of both hyperparameters ($C_{1}$ for feature selection, $C_{2}$ for machine learning model production) were optimized through a grid search running over a large range of combinations. The C-parameter associated with the feature selection phase, $C_{1}$, was varied between 0.005 and 0.05. This ensured that the number of retained features was always <20 to minimize the risk of over-fitting the data during the model development phase, during which $C_{2}$ was varied from 0.1 to 5.

Each combination of hyperparameters $\left(C_{1},C_{2}\right)$ led to a different machine-learning model. A training/validation process was utilized to find the parameters leading to optimal predictive performances, i.e., to select those parameters that led to the smallest number of false positive and false negative predictions. Importantly, this needed to be done ensuring the final models generalized well to new data, namely, to ensure an optimal balance was reached between under-fitting and over-fitting the spectral data. This was achieved during a training/validation phase using a fivefold cross-validation technique applied on 60% of the *ex vivo* dataset, retaining the remaining 40% to evaluate model performances on an independent holdout data subset.

For models II and III, the performance during the training/validation phase (using 60% of the data) was assessed through a receiver-operating-characteristic (ROC) analysis. An ROC curve (x-axis, specificity; y-axis, 1 - sensitivity) was generated and the selected final model had the largest sensitivity and specificity values. Here, this corresponded to the point on the ROC curve that had the shortest distance to the upper-left corner. The 2×2 confusion matrix associated with the ROC analysis was also produced to showcase the number of false positive/negative and true positive/negative instances. The optimal machine-learning model was then applied directly onto the holdout dataset (40% of the *ex vivo* dataset) and the resulting predictive performances were reported using another confusion matrix.

All data acquired during the *in situ* and *in vivo* experiments were then used as another way to test the models using independent data to assess their generalizability. All data from those experiments were applied to models II and III, leading to tissue classes prediction, along with a quantitative measure of the probability of association (an output from the SVM analysis) with each class ranging from 0 to 1.^37^ For example, in the case of model II, a probability of association close to 1 indicates that the model predicted, with high confidence, that the Raman spectral fingerprint was associated with spinal cord tissue, while a value closer to 0 indicated that the model confidently associated the measurement to bone.

ROC analyses do not lend themselves to performance analyses in the case of multi-class (i.e., more than two classes) models. Hence, the performance for Model I was assessed using a confusion matrix $M_{ij}$, where i and j each run from 1 to 6. The diagonal elements of the matrix report the number of correct predictions for each class, while the off-diagonal elements tabulate the number of incorrect model predictions. Specifically, off-diagonal elements (i,j) with i≠j correspond to the number of predictions associating a Raman spectral fingerprint to tissue category i that should have been associated with tissue category j, and *vice-versa*. As in the performance assessment of Models II and III, two confusion matrices were produced, one for the training/validation phase and one from the testing phase. For the latter, the six-class tissue discrimination model was applied directly to the hold-out set comprising 40% of the *ex vivo* dataset. The analysis associated with applying model I to the *in situ* and *in vivo* datasets is not presented here for conciseness.

## 3 Results

### 3.1 Band Assignment and Raman Spectral Fingerprint Interpretation

**Fig. 4:**
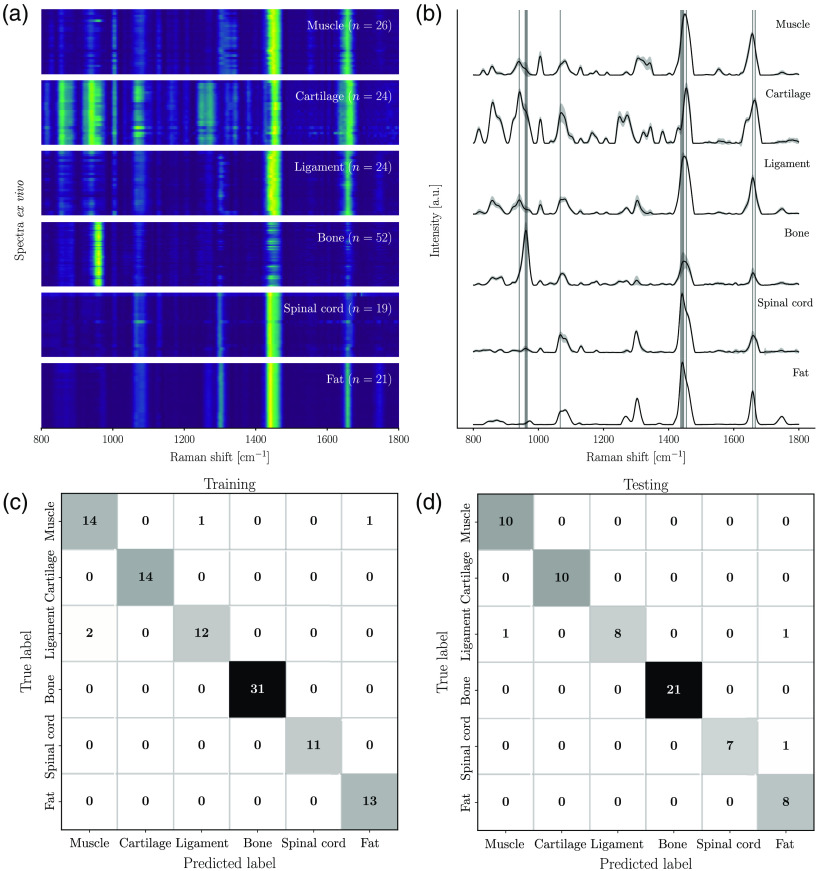
Multi-class *ex vivo* tissue classification. (a) Spectrogram showing the entire *ex vivo* dataset, demonstrating qualitatively the consistency of specific tissue spectra across all measurements. (b) Average spectra per tissue type, with the corresponding variance computed for each spectral bin across all measurements. Vertical gray bands represent the features selected for machine-learning classification. The confusion matrices are shown in (c) and (d) for the training and testing sets, respectively.

Figure 4(a) shows the complete *ex vivo* dataset as a spectrogram, individual SNV-normalized Raman spectra, vertically stacked and grouped by tissue type. The intensity at each wavenumber is represented by a false-color scale, ranging from dark blue (minimum) to light green (maximum). Figure 4(b) shows the average spectrum for each tissue category, together with the variance across all measurements, computed for each spectral bin. Table 1 summarizes the visually detectable Raman bands for all tissue types that are associated with known molecular vibrational modes. A tentative association is made to families of biomolecules, and the relative intensity for each peak center is shown. The relative intensity (across all tissue types) for each peak is indicated using a scale from 1 to 4 (represented by asterisks: *, **, *** or ****) based on the results of univariate statistical analysis (student t-test) applied to the average peak intensities. Since each of the tissues of interest has distinct molecular composition, the distinguishing Raman signatures can be clearly seen.

Looking at the muscle column in Table 1, most bands were directly linked to proteins and amino acids and were in good agreement since proteins are the most important component of striated skeletal muscle.^43^ The peaks at 1131 and 1448 to $1451\;{\mathrm{cm}}^{-1}$ encompass fatty acids and lipids; the presence of which is expected since intramuscular fat accumulates both within (intramyocellular) and surrounding (extramyocellular) muscle fibers. For the spinal cord the largest peak is at 1439 to $1441\;{\mathrm{cm}}^{-1}$ corresponding to DNA/RNA, protein (amide I), and lipids. The aggregation of these different molecular species represents the distribution of proteins and genetic information in nerve fiber bundles within the spinal cord. Lipids also often accompany proteins, since the spinal cord comprises both white and gray matter. The myelin sheath surrounding the nerve fibers that compose the spinal cord also has high lipid content (70% to 80%).^44^ Interestingly, in the fat tissues, the highest peak is at $1305\;{\mathrm{cm}}^{-1}$, assignment to collagen. Adipocytes (fat cells) produce mainly collagen that aids cell adhesion, differentiation, and wound healing.^45^ Most of the other peaks relate to lipids, which confirms the fat tissues composition.

Connective tissue in the table groups cartilage and ligament together, based on the main collagen constituent with the highest peak at $817\;{\mathrm{cm}}^{-1}$, as well as peaks at 857, 1067 to 1069, 1303, and 1448 to $1451\;{\mathrm{cm}}^{-1}$. The material strength and biological properties of articular cartilage depend heavily on its unique and extensively cross-linked extracellular collagen network and characteristic fibrillar organization that varies with tissue depth and cellular proximity. Ligaments are generally composed of ground substance, collagen (mainly types I and III) with minimal elastin fibers, i.e., the building blocks are collagen fibers. Another high peak at $1641\;{\mathrm{cm}}^{-1}$ is assigned to water,^46^ which is an important component of articular cartilage, contributing up to 80% of wet weight. Part of this water is linked to the intrafibrillar space within the collagen.^47^ Cartilage also has a high peak at 959 to $965\;{\mathrm{cm}}^{-1}$ for calcium hydroxyapatite corresponding to mineral calcium, deposits of which can be found in articular cartilage. Overall, connective tissue includes all the peaks selected, since the properties of connective tissue reside in the amount, type, and arrangement of abundant extracellular matrix. By contrast, the biological properties of tissues such as spinal cord, fat, or muscle depend mostly on their cellular elements.^48^

Finally, bone has its highest peak at 959 to $965\;{\mathrm{cm}}^{-1}$, assigned to calcium hydroxyapatite. Bone comprises both a mineral (inorganic) and an organic phase. Calcium hydroxyapatite is the main component of the former, which makes up ∼60% of the tissue.^49^

### 3.2 *Ex Vivo* Multi-Class Tissue Classification Model

Visual inspection of the individual Raman spectra [Fig. 4(a)] and their average for each tissue type [Fig. 4(b)] shows that the spectral fingerprints acquired on freshly excised specimens (i.e., the *ex vivo* spectral dataset) have clearly distinguishable features enabling tissue discrimination. As the mineral peak at $961\;{\mathrm{cm}}^{-1}$ is unique to trabecular and cortical bone, it can be easily distinguished from soft tissues. Although fat and spinal cord show similarities, they can be separated according to the phenylalanine peak at $1004\;{\mathrm{cm}}^{-1}$. Muscle, cartilage, and ligament tissues are also distinguishable, given that the peak around $1451\;{\mathrm{cm}}^{-1}$ is weak in cartilage, while the phenylalanine peak at $1004\;{\mathrm{cm}}^{-1}$ helps to further differentiate between muscle and ligament.

The vertical gray regions in Fig. 4(b) represent the spectral bands that were picked up by the feature-selection algorithm in building the six-class machine-learning model (Model I). A total of 13 spectral bands were required to achieve an accuracy >96% in both the training/validation and the testing. This is shown in the confusion matrices for the training/validation phase [Fig. 4(c)] and the testing phase [Fig. 4(d)], where only 3 of 98 and 2 of 64 spectra were misclassified, respectively.

### 3.3 *In Vivo* and *In Situ* Testing of the Two-class Soft Tissue Detection Models

**Fig. 5:**
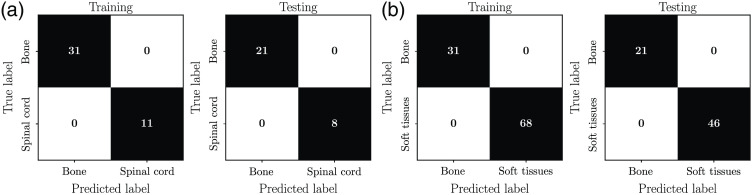
Two-class *ex vivo* tissue classification models. Confusion matrices quantifying model performance on training and testing sets: (a) model II – bone versus spinal cord and (b) model III – bone versus soft tissues.

The confusion matrices from the training/validation and testing phases associated with model II (bone versus spinal cord) are shown in Fig. 5(a). The corresponding confusion matrices associated with Model III (bone versus soft tissues) are shown in Fig. 5(b).

**Fig. 6:**
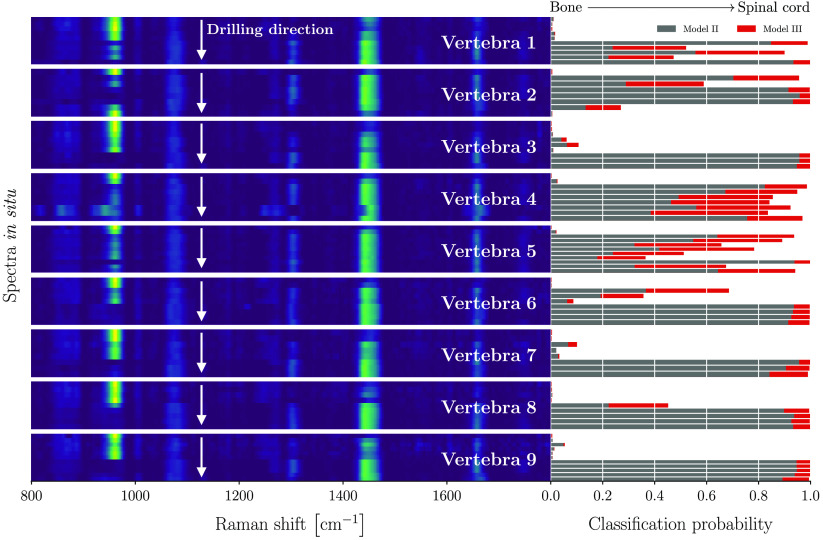
Two-class soft tissue detection models applied to *in situ* data: model II (bone versus spinal cord), model III (bone versus soft tissues). Raman fingerprint spectrograms are shown for the entire *in situ* dataset, for each of the nine vertebrae, qualitatively demonstrating the transition from bone to spinal cord through the disappearance of the mineral peak at $961\;{\mathrm{cm}}^{-1}$ as the probe approaches soft tissue. The classification probability for each model and for each individual spectrum is shown in gray for model II and in red for model III. A value closer to 1 associates the measurement to bone, while a value closer to 0 associates it to soft tissue.

**Fig. 7:**
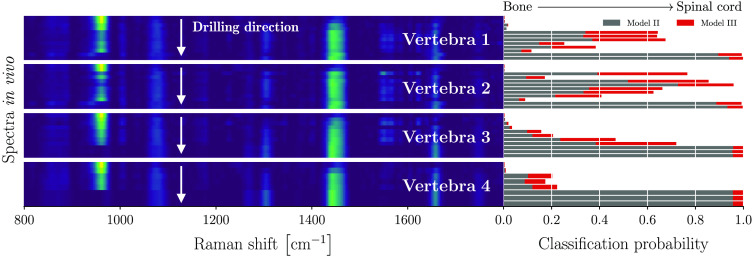
Two-class soft tissue detection models applied to the *in vivo* data: model II (bone versus spinal cord) and model III (bone versus soft tissues). Raman fingerprint spectrograms are shown for the entire dataset for each of the four vertebrae, qualitatively demonstrating the transition from bone to spinal cord through the disappearance of the mineral peak at $961\;{\mathrm{cm}}^{-1}$ as the RS probe approaches soft tissue. The classification probabilities for each model and for each individual spectrum are shown in gray for model II and in red for model III.

The models performed with 100% sensitivity during both the training and testing phases. These two-class models were then applied to the *in situ* dataset (Fig. 6) and the *in vivo* dataset (Fig. 7) to evaluate how well they generalized to situations closer to real-world surgical orthopedic procedures. Both models II and III used only two features, namely the mineral peak at $961\;{\mathrm{cm}}^{-1}$ and the amide I peak at $1441\;{\mathrm{cm}}^{-1}$.

Figure 6 shows the Raman spectrograms associated with all vertebrae for which *in situ* measurements were made. The y-axis in these plots represents the drilling direction, i.e., the axis along which the Raman measurements were made, starting from a bone region to anatomical areas associated with soft tissue, i.e., spinal cord. The transition from bone to spinal cord can be appreciated through visual inspection, namely the disappearance of the mineral peak at $961\;{\mathrm{cm}}^{-1}$ and the concomitant gradual appearance of the amide bands.

The spectrograms also consistently demonstrate a pattern of initial bone followed by a well-differentiated soft tissue signature, mostly resembling that of spinal cord. This is confirmed by the numerical values associated with the confidence-of-association to a tissue class when using model III (bone versus soft tissue). All measurements with a strong mineral peak and low intensity amide bands are associated with probabilities of associated to bone close to 1 (red bars in Fig. 6). Similar trends are observed when applying model II (bone versus spinal cord) to the same dataset (gray bars in Fig. 6). However, the numerical values associated with the probability of association to bone, although consistently larger than 0.5, are smaller than those obtained in model III. The higher confidence using model III may be due to the fact it was trained using a more heterogeneous dataset that better reflects the anatomical environment moving from bone to spinal cord.

Figure 7 shows that similar conclusions are reached when applying Models II and III to the *in vivo* measurements. However, these data have higher inter-measurement variances compared with the *ex vivo* and *in situ* measurements, likely due to interference from bleeding that both introduced additional materials and may have increased light attenuation even with the use of a saline flush during drilling. Despite this, the distinctive features of bone and spinal cord tissues still allow unambiguous identification of the bone–spinal cord interface.

## 4 Discussion and Future Directions

Demands of the orthopedic surgical environment often require fast, reliable, and actionable guidance with consistent performance in every case. Here, tissue measurements took up to several seconds to complete in some cases. Differentiation of bone and spinal cord was typically achieved in ∼1  s, with larger measurement times required in, e.g., muscle identification. Detection times of this order may be detrimental to real-world clinical integration. In fact, the requirement for surgical deployment would require spectral fingerprints consistently acquired in near real-time, for example, <∼100  ms. Modifications to the current fiberoptic system are feasible that could increase the amount of collected light to reduce interrogation times while maintaining good signal-to-noise in the spectra. This could be achieved multiple ways, including, for example, using a spectrophotometer with increased light sensitivity (e.g., HT rather than EH spectrometer from EmVision LLC) and a more powerful laser, within the maximum permissible exposure limits to ensure minimal tissue damage from increased temperature (e.g., <43°C).^50^ Other more stringent safety standards as set by ANSI Z136.3 Laser Safety in Healthcare for skin and ocular exposition could be addressed through user guidelines and using safety goggles.

Another factor that may allow significant reduction of the tissue interrogation time is determination of the minimal SNR thresholds required for each tissue type to achieve real-time tissue classification. This was not a consideration in the above proof-of-principle studies in which the integration time and number of accumulations were optimized to ensure that the spectra were shot-noise limited with minimal contribution from stochastic photonic noise. This led to spectral fingerprints for all tissue types with almost no stochastic noise. However, the spectral quality achieved likely surpassed that required to distinguish bone from soft tissue in the real-world, where only the most active Raman bands need to be detected, including the mineral peak and amide bands.

As a result, if proper mechanical integration of the optical fibers with current trocar devices is achieved, then minimal alterations to the fiberoptic technology and the data acquisition and laser safety protocols could be made to that the RS guidance is acquired seamlessly with minimum impact on the surgical workflow. If so, then a significant decrease in safe pedicle screw placement could be achieved, reducing the current dependence on slow, cumbersome, and ionizing intraoperative imaging. A further aspect not considered in this study was the impact of depth sampling on the potential depth accuracy of RS in orthopedic surgery. The diffuse nature of tissue light transport in the NIR means that the effective sampling depth beyond the probe tip depends on the albedo, which varies between tissue types in the range ∼100 to 600  μm.^51^ The impact of this tissue-dependent sensing depth needs to be investigated further.

The machine-learning approach used here detected the bone/spinal canal interface successfully in all cases. However, increased variance was observed during the drilling measurements *in situ* and *in vivo*. While it is likely that some of this variance can be attributed to physical mixing of tissue types in the drilling, it may explain the increased uncertainty in the model predictions near the cord/bone interface. Furthermore, nerve roots, which were not included in the analysis since their location was unknown, may also have contributed to this uncertainty, even when drilling far from the spinal cord. This may be a challenge in clinical translation.

Another important aspect of the current study was the demonstration that the spectral fingerprint of multiple tissue types could be detected around the spine, not only under ideal *ex vivo* conditions but also under realistic surgical conditions, i.e., *in vivo*. This paves the way to other potential applications such as detection of bone abnormalities associated with osteoporosis, as well as guidance during osteoblastoma and osteosarcoma surgeries to reduce risk of recurrence due to incomplete resection. ^52^^–^^55^

## 5 Conclusions

We have shown that RS holds potential for guiding orthopedic surgical procedures where knowledge of the tissue type is critical. Using supervised machine learning binary classification on RS spectra during bone drilling identified critical structures such as the bone/spinal canal interface rapidly and with high accuracy. Even when stressed to differentiate six types of tissue found in the vertebra, the model performed at higher than 96% accuracy. The development of an “intelligent” drill bit based on RS could then reduce the currently high inter-surgeon variability during intra-pedicular drilling and reduce or replace expensive, time-consuming, and indirect radiological image-guidance procedures.

## Code, Data, and Materials Availability

The data and materials information that support the findings of this study are available from the corresponding author upon reasonable request.

## References

[1] Reisener M.-J. et al., “Trends in lumbar spinal fusion: a literature review,” J. Spine Surg. 6(4), 752–761 (2020). 10.21037/jss-20-492 33447679 PMC7797794

[2] Longo U. G. et al., “Augmented reality, virtual reality and artificial intelligence in orthopedic surgery: a systematic review,” Appl. Sci. 11(7), 3253 (2021). 10.3390/app11073253

[3] Pokorny G. et al., “Minimally invasive versus open surgery for degenerative lumbar pathologies: a systematic review and meta-analysis,” Eur. Spine J. 31(10), 2502–2526 (2022). 10.1007/s00586-022-07327-3 35871660 PMC9308956

[r4] Gaudin D. et al., “Considerations in spinal fusion surgery for chronic lumbar pain: psychosocial factors, rating scales, and perioperative patient education—a review of the literature,” World Neurosurg. 98, 21–27 (2017). 10.1016/j.wneu.2016.10.124 27810456

[5] Ponkilainen V. T. et al., “National trends in lumbar spine decompression and fusion surgery in Finland, 1997–2018,” Acta Orthop. 92(2), 199–203 (2021). 10.1080/17453674.2020.1839244 33106074 PMC8158253

[6] Vardiman A. B. et al., “Does the accuracy of pedicle screw placement differ between the attending surgeon and resident in navigated robotic-assisted minimally invasive spine surgery?” J. Robot. Surg. 14(4), 567–572 (2020). 10.1007/s11701-019-01019-9 31542860 PMC7347677

[r7] Flynn J. M. Sakai D. S., “Improving safety in spinal deformity surgery: advances in navigation and neurologic monitoring,” Eur. Spine J. 22(S2), 131–137 (2013). 10.1007/s00586-012-2360-6 PMC3616474 22614688

[8] Kosmopoulos V. et al., “Observer reliability in evaluating pedicle screw placement using computed tomography,” Int. Orthop. 31(4), 531–536 (2007). 10.1007/s00264-006-0230-8 16967277 PMC2267647

[9] Torii Y. et al., “Screw insertion time, fluoroscopy time, and operation time for robotic-assisted lumbar pedicle screw placement compared with freehand technique,” Cureus 14, e25039 (2022). 10.7759/cureus.25039 35719818 PMC9199567

[10] Mulconrey D. S., “Fluoroscopic radiation exposure in spinal surgery: *in vivo* evaluation for operating room personnel,” Clin. Spine Surg. Spine Publ. 29(7), E331–E335 (2016). 10.1097/BSD.0b013e31828673c1 27171657

[r11] Gill S. et al., “Biomechanically constrained groupwise ultrasound to CT registration of the lumbar spine,” Med. Image Anal. 16(3), 662–674 (2012). 10.1016/j.media.2010.07.008 21126904

[12] Waschke A. et al., “CT-navigation versus fluoroscopy-guided placement of pedicle screws at the thoracolumbar spine: single center experience of 4,500 screws,” Eur. Spine J. 22(3), 654–660 (2013). 10.1007/s00586-012-2509-3 23001415 PMC3585623

[13] Wang V. Y. et al., “Free-hand thoracic pedicle screws placed by neurosurgery residents: a CT analysis,” Eur. Spine J. 19(5), 821–827 (2010). 10.1007/s00586-010-1293-1 20135332 PMC2899961

[14] Romero-Muñoz L. M. et al., “Neurological injury as a complication of spinal surgery: incidence, risk factors, and prognosis,” Spinal Cord 58(3), 318–323 (2020). 10.1038/s41393-019-0367-0 31619752

[15] Proietti L. et al., “Complications in lumbar spine surgery: a retrospective analysis,” Indian J. Orthop. 47(4), 340–345 (2013). INJOAU 10.4103/0019-5413.114909 23960276 PMC3745686

[16] Swamy A. et al., “Diffuse reflectance spectroscopy for breach detection during pedicle screw placement: a first *in vivo* investigation in a porcine model,” Biomed. Eng. OnLine 19(1), 47 (2020). 10.1186/s12938-020-00791-2 32532305 PMC7291697

[r17] Liu Y. et al., “Monitoring the reduced scattering coefficient of bone tissues on the trajectory of pedicle screw placement using near-infrared spectroscopy,” J. Biomed. Opt. 19(11), 117002 (2014). JBOPFO 1083-3668 10.1117/1.JBO.19.11.117002 25371980

[18] Gonzalez E. A. Jain A. Bell M. A. L., “Combined ultrasound and photoacoustic image guidance of spinal pedicle cannulation demonstrated with intact *ex vivo* specimens,” IEEE Trans. Biomed. Eng. 68(8), 2479–2489 (2021). IEBEAX 0018-9294 10.1109/TBME.2020.3046370 33347403 PMC8345233

[19] Qureshi R. et al., “Perioperative management of blood loss in spine surgery,” Clin. Spine Surg. Spine Publ. 30(9), 383–388 (2017). 10.1097/BSD.0000000000000532 28338491

[20] Cordero E., “*In-vivo* Raman spectroscopy: from basics to applications,” J. Biomed. Opt. 23(7), 071210 (2018). JBOPFO 1083-3668 10.1117/1.JBO.23.7.071210 29956506

[r21] DePaoli D. et al., “Rise of Raman spectroscopy in neurosurgery: a review,” J. Biomed. Opt. 25(5), 050901 (2020). JBOPFO 1083-3668 10.1117/1.JBO.25.5.050901 32358930 PMC7195442

[r22] Movasaghi Z. Rehman S. Rehman I. U., “Raman spectroscopy of biological tissues,” Appl. Spectrosc. Rev. 42(5), 493–541 (2007). APSRBB 0570-4928 10.1080/05704920701551530

[r23] Aubertin K. et al., “Mesoscopic characterization of prostate cancer using Raman spectroscopy: potential for diagnostics and therapeutics,” BJU Int. 122(2), 326–336 (2018). BJINFO 1464-410X 10.1111/bju.14199 29542855

[24] Jermyn M. et al., “Intraoperative brain cancer detection with Raman spectroscopy in humans,” Sci. Transl. Med. 7(274), 274ra19–274ra19 (2015). STMCBQ 1946-6234 10.1126/scitranslmed.aaa2384 25673764

[25] Shaikh R. et al., “Raman spectroscopy is sensitive to biochemical changes related to various cartilage injuries,” J. Raman Spectrosc. 52(4), 796–804 (2021). JRSPAF 0377-0486 10.1002/jrs.6062

[26] Pavlou E. et al., “Raman spectroscopy for the assessment of osteoarthritis,” Ann. Jt. 3, 83–83 (2018). 10.21037/aoj.2018.09.10

[27] Buckley K. et al., “Towards the *in vivo* prediction of fragility fractures with Raman spectroscopy: prediction of fragility fractures with Raman spectroscopy,” J. Raman Spectrosc. 46(7), 610–618 (2015). JRSPAF 0377-0486 10.1002/jrs.4706 27546955 PMC4976623

[28] Fraulob M. et al., “Multimodal characterization of the bone-implant interface using Raman spectroscopy and nanoindentation,” Med. Eng. Phys. 84, 60–67 (2020). MEPHEO 1350-4533 10.1016/j.medengphy.2020.07.013 32977923

[29] Fosca M. et al., “Raman spectroscopy in skeletal tissue disorders and tissue engineering: present and prospective,” Tissue Eng. Part B Rev. 28(5), 949–965 (2022). 10.1089/ten.teb.2021.0139 34579558 PMC9587790

[30] Stevens O. et al., “Developing fibre optic Raman probes for applications in clinical spectroscopy,” Chem. Soc. Rev. 45(7), 1919–1934 (2016). CSRVBR 0306-0012 10.1039/C5CS00850F 26956027

[31] Kim W. et al., “Lensless, ultra-wideband fiber optic rotary joint for biomedical applications,” Opt. Lett. 41(9), 1973 (2016). OPLEDP 0146-9592 10.1364/OL.41.001973 27128052 PMC6731063

[32] Desroches J. et al., “Characterization of a Raman spectroscopy probe system for intraoperative brain tissue classification,” Biomed. Opt. Express 6(7), 2380 (2015). BOEICL 2156-7085 10.1364/BOE.6.002380 26203368 PMC4505696

[33] Busscher I. et al., “Comparative anatomical dimensions of the complete human and porcine spine,” Eur. Spine J. 19(7), 1104–1114 (2010). 10.1007/s00586-010-1326-9 20186441 PMC2900026

[34] David S. et al., “*In situ* Raman spectroscopy and machine learning unveil biomolecular alterations in breast cancer,” J. Biomed. Opt. 28(3), 036009 (2023). 10.1117/1.JBO.28.3.036009 37009577 PMC10062385

[35] Lemoine É. et al., “Feature engineering applied to intraoperative *in vivo* Raman spectroscopy sheds light on molecular processes in brain cancer: a retrospective study of 65 patients,” The Analyst 144(22), 6517–6532 (2019). ANALAO 0003-2654 10.1039/C9AN01144G 31647061

[36] Jermyn M. et al., “Highly accurate detection of cancer* in situ* with intraoperative, label-free, multimodal optical spectroscopy,” Cancer Res. 77(14), 3942–3950 (2017). CNREA8 0008-5472 10.1158/0008-5472.CAN-17-0668 28659435

[37] David S. et al., “Multispectral label-free Raman spectroscopy can detect ovarian and endometrial cancer with high accuracy,” J. Biophotonics 15(2, e202100198 2022). 10.1002/jbio.202100198 34837331

[38] Picot F. et al., “Image-guided Raman spectroscopy navigation system to improve transperineal prostate cancer detection. Part 1: Raman spectroscopy fiber-optics system and in situ tissue characterization,” J. Biomed. Opt. 27(9), 095003 (2022). JBOPFO 1083-3668 10.1117/1.JBO.27.9.095003 36045491 PMC9433338

[r39] Aubertin K. et al., “Combining high wavenumber and fingerprint Raman spectroscopy for the detection of prostate cancer during radical prostatectomy,” Biomed. Opt. Express 9(9), 4294 (2018). BOEICL 2156-7085 10.1364/BOE.9.004294 30615702 PMC6157766

[40] Grajales D. et al., “Image-guided Raman spectroscopy navigation system to improve transperineal prostate cancer detection. Part 2: *in-vivo* tumor-targeting using a classification model combining spectral and MRI-radiomics features,” J. Biomed. Opt. 27(9), 095003 (2022). JBOPFO 1083-3668 10.1117/1.JBO.27.9.095004 36085571 PMC9459023

[41] Sheehy G. et al., “Open-sourced Raman spectroscopy data processing package implementing a baseline removal algorithm validated from multiple datasets acquired in human tissue and biofluids,” J. Biomed. Opt. 28(2), 025002 (2023). JBOPFO 1083-3668 10.1117/1.JBO.28.2.025002 36825245 PMC9941747

[42] Plante A. et al., “Dimensional reduction based on peak fitting of Raman micro spectroscopy data improves detection of prostate cancer in tissue specimens,” J. Biomed. Opt. 26(11), 116501 (2021). JBOPFO 1083-3668 10.1117/1.JBO.26.11.116501 34743445 PMC8571651

[43] Makovický P. Makovický P. Jílek F., “Short review of some properties of muscular proteins,” Cesk Fysiol. 57(1), 10–14 (2008). 18630139

[44] Poitelon Y. Kopec A. M. Belin S., “Myelin fat facts: an overview of lipids and fatty acid metabolism,” Cells 9(4), 812 (2020). 10.3390/cells9040812 32230947 PMC7226731

[45] Ruiz-Ojeda F. J. et al., “Extracellular matrix remodeling of adipose tissue in obesity and metabolic diseases,” Int. J. Mol. Sci. 20(19), 4888 (2019).1422-0067 10.3390/ijms20194888 31581657 PMC6801592

[46] Sergeeva A. V. et al., “Infrared and Raman spectroscopy of ammoniovoltaite, (NH4)2Fe2+5Fe3+Al3(SO4)12(H2O)18,” Minerals 10(9), 781 (2020). 10.3390/min10090781

[47] Sophia Fox A. J. Bedi A. Rodeo S. A., “The basic science of articular cartilage: structure, composition, and function,” Sports Health Multidiscip. Approach 1(6), 461–468 (2009). 10.1177/1941738109350438 PMC3445147 23015907

[48] Culav E. M. Clark C. H. Merrilees M. J., “Connective tissues: matrix composition and its relevance to physical therapy,” Phys. Ther. 79(3), 308–319 (1999). POTPDY 10.1093/ptj/79.3.308 10078774

[49] Feng X., “Chemical and biochemical basis of cell-bone matrix interaction in health and disease,” Curr Chem Biol. 3(2), 189–196 (2009). 10.2174/187231309788166398 20161446 PMC2790195

[50] Daoust F. et al., “Clinical prototype Raman imaging system for intraoperative machine-learning-based molecular tissue characterization,” Analyst 148(9), 1991–2001 (2023). ANALAO 0003-2654 37038988 10.1039/d2an01946a

[51] Akbarzadeh A. et al., “Experimental validation of a spectroscopic Monte Carlo light transport simulation technique and Raman scattering depth sensing analysis in biological tissue,” J. Biomed. Opt. 25(10), 105002 (2020). JBOPFO 1083-3668 10.1117/1.JBO.25.10.105002 33111509 PMC7720906

[52] Spraker-Perlman H. L. et al., “Factors influencing survival after recurrence in osteosarcoma: a report from the Children’s Oncology Group,” Pediatr. Blood Cancer 66(1), e27444 (2019). 10.1002/pbc.27444 30255612 PMC6249072

[r53] Kempf-Bielack B. et al., “Osteosarcoma relapse after combined modality therapy: an analysis of unselected patients in the Cooperative Osteosarcoma Study Group (COSS),” J. Clin. Oncol. 23(3), 559–568 (2005). JCONDN 0732-183X 10.1200/JCO.2005.04.063 15659502

[r54] Honda A. et al., “Recurrent lumbar-origin osteoblastoma treated with multiple surgery and carbon ion radiotherapy: a case report,” BMC Musculoskelet. Disord. 21(1), 321 (2020). 10.1186/s12891-020-03349-4 32443969 PMC7245031

[55] Mesfin A. et al., “Can osteoblastoma evolve to malignancy? A challenge in the decision-making process of a benign spine tumor,” World Neurosurg. 136, 150–156 (2020). 10.1016/j.wneu.2019.11.148 31809897

